# A simple and robust LC-ESI single quadrupole MS-based method to analyze neonicotinoids in honey bee extracts

**DOI:** 10.1016/j.mex.2019.09.038

**Published:** 2019-10-17

**Authors:** Melanie Allgaier, Julia M. Halder, Jens Kittelberger, Bernhard Hauer, Bernd A. Nebel

**Affiliations:** Institute of Biochemistry and Technical Biochemistry, Department of Technical Biochemistry, University of Stuttgart, Allmandring 31, 70569 Stuttgart, Germany

**Keywords:** Analyzing neonicotinoids in honey bee extracts by LC-ESI single-quadrupole MS, Honey bees, Neonicotinoids, Thiacloprid, Flupyradifurone, LC–MS, Single quadrupole, Sample preparation, QuEChERS

## Abstract

Over the past years, neonicotinoids such as thiacloprid and flupyradifurone have gained considerable scientific and public interest. These molecules used as active compounds in pesticides are known due to cause drastic negative long-time effects on pollinators and even human health. Therefore, determining trace amounts of neonicotinoid in different environmental matrices by liquid chromatography coupled with mass selective detectors (LC—MS/MS or LC-Q-TOF/MS) has become an important methodology. However, not every scientific group has unlimited access to high-resolution mass-selective detectors (*e.g.*, MS/MS). It becomes more apparent that the analytics of neonicotinoids are already a global issue. Research groups and organizations with a limited financial budget often depend on using cheap and robust equipment to do their analytical work. We demonstrate a single-quadrupole (Q) MS-based method with single-class residue methods (SRMs) for the analysis of neonicotinoids, applicable without the requirement of a high-end MS system. For an adequate sample clean-up strategy, QuEChERS (Quick, Easy, Cheap, Efficient, Rugged, safe) extraction and purification methods were modified and applied to eliminate residual matrix after honey bee extraction steps to analyze thiacloprid and flupyradifurone.

• Simple liquid chromatography electro-spray ionization (LC-ESI) single-quadrupole mass selective (MS) method for neonicotinoid analysis.
• Efficient sample pretreatment by a modified QuEChERS extraction and purification method.
• Limit of detection (LOD) and limit of quantification (LOQ) for thiacloprid was 19.72 ng g^−1^ and 7.61 ng g^−1^, for flupyradifurone 65.73 ng g^−1^ and 25.36 ng g^−1^, respectively.

**Specification Table**

**Table:** 

Subject Area:	Agricultural and Biological Sciences
More specific subject area:	Analytic and sample pre-treatment
Method name:	Analyzing neonicotinoids in honey bee extracts by LC-ESI single-quadrupole MS
Name and reference of original method:	Unbuffered QuEChERS sample were pre-treated according to reference [15] with further modifications described in this paper.
Resource availability:	LC-MS method is not based on an original method.

## Method details

### Safety protocol

Neonicotinoids are toxic and harmful to the environment and human being and thus should be carefully handled

### Reagents and standards

Analytical standards of thiacloprid (C17451000) and flupyradifurone (37,050) were obtained from Dr. Ehrenstorfer (Dr. Ehrenstorfer GmbH, Augsburg, Germany) and Sigma-Aldrich (Sigma-Aldrich Chemie GmbH, Taufkirchen, Germany), respectively. Acetonitrile (ACN), CHROMASOLV™ for high liquid pressure chromatography (HPLC) with gradient grade (34851, Honeywell Riedel-de Haën™, Seelze, Germany) and bidest. ultra-pure water from a Milli-Q system (Merck KGaA, Darmstadt, Germany) were used for sample preparation as well as for LC–MS analysis. Formic acid puriss. (27,001) was purchased from Honeywell (Honeywell Riedel-de Haën™, Seelze, Germany) and sodium chloride, (≥99%, Ph. Eur., USP P029.3) was bought from Carl Roth (Carl Roth GmbH + Co. KG, Karlsruhe, Germany). Anhydrous magnesium sulfate (M7506) was also obtained from Sigma-Aldrich (Sigma-Aldrich Chemie GmbH, Taufkirchen, Germany). The used solid phase extraction (SPE) material and the grinding media were obtained in a dispersive 2 mL (universal kit 5982-0028CH, Agilent Technologies Sales & Services GmbH & Co. KG, Waldbronn, Germany).

Note: Availability of standard laboratory and chromatographically equipment is assumed.

### Sample collection and pre-treatment

**Fig. 1:**
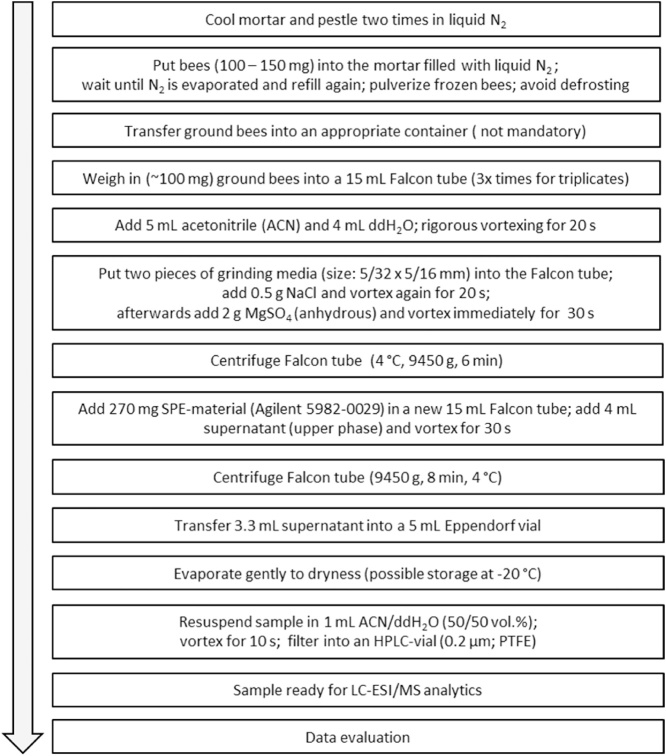
Honey bee sample clean-up procedure based on a modified QuEChERS method.

Thiacloprid- as well as flupyradifurone-contaminated honey bees were made available by our collaboration partner, Institute of Biology and Neurobiology, Freie Universtät Berlin, Germany. Bees were fed in lab experiments with contaminated sugar solutions, and stored at −80 °C. All samples were extracted according to the method demonstrated in Fig. 1.

**Fig. 2:**
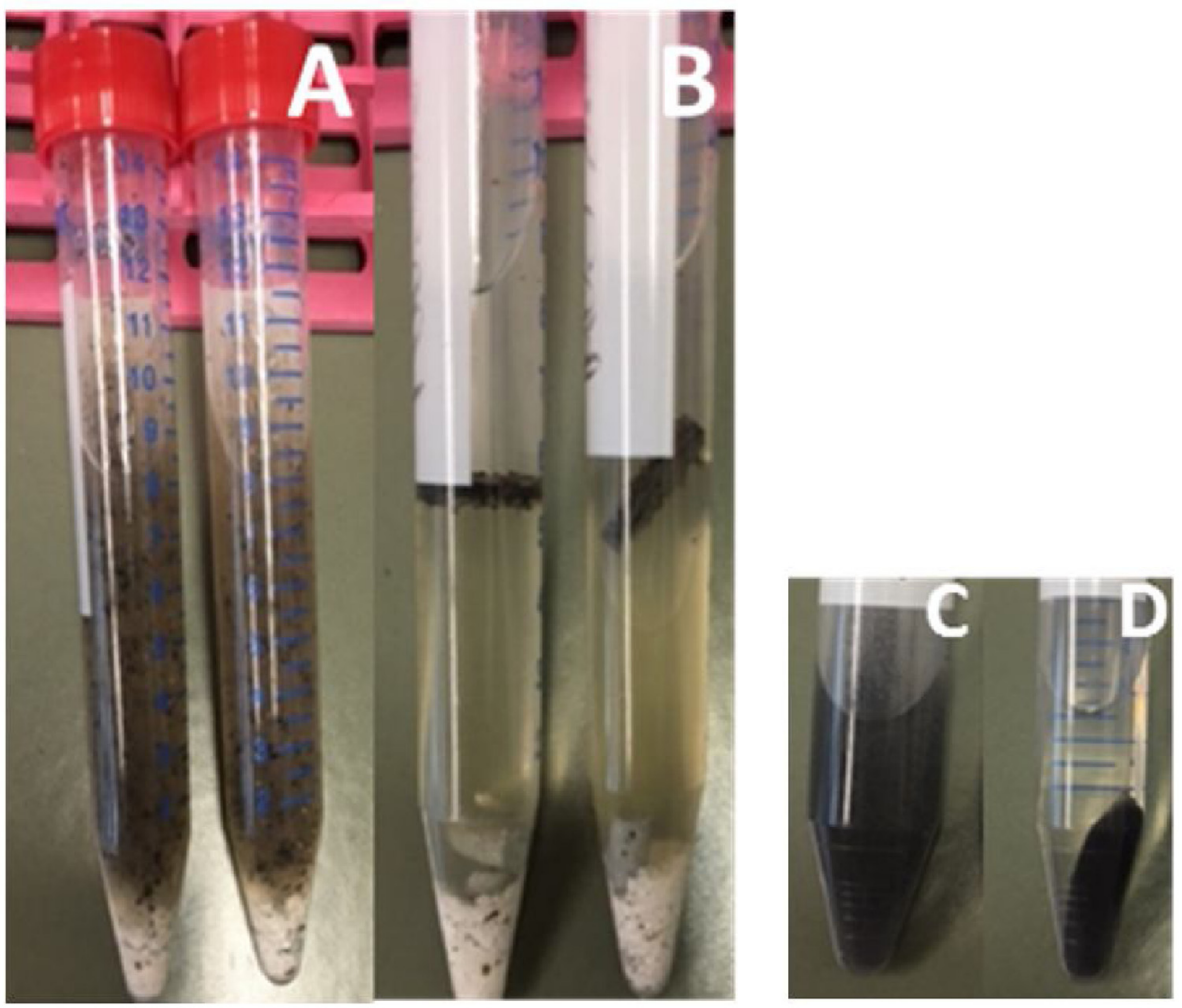
Honey bee sample clean-up procedure based on a modified QuEChERS (Quick, Easy, Cheap, Efficient, Rugged, Safe) method. Picture A shows a 15 mL Falcon tube containing bee sample with water, acetonitrile, NaCl, MgSO_4_, and grinding media (duplicate). Picture B illustrates the phase separation after centrifugation. The supernatant from B was transferred, and SPE material was added (picture C). Picture D represents the clear extract of the supernatant after centrifugation.

A few modifications were made to the recommended standard QuEChERS (Quick, Easy, Cheap, Efficient, Rugged, Safe) extraction procedure to obtain more accurate results. To ensure a proper phase separation during the extraction procedure, it was critical to avoid any defrosting of the bee samples during and immediately after the initial grinding process (grind the sample as fine and homogenous as possible). Therefore, the mortar and pestle were cooled down twice by filling the mortar with liquid nitrogen (N_2)_ and waiting until the N_2_ had fully evaporated, before filling it up a third time and directly adding the bees, which were stored at −80 °C. After the addition of any new ingredient in the extraction step, rigorous shaking for 20 and 30 s, respectively, using a vortex was essential to achieve high extraction rates. Longer vortexing did not increase the extraction rate any further. Usage of anhydrous magnesium sulfate (MgSO_4_) avoids clumping. After addition of MgSO_4_, instant vortexing had to be done to avoid precipitation effects that might affect phase separation. This step is very time critical and crucial for the success of the experiment. In this study, bulk reservoirs of sodium chloride (NaCl) and MgSO_4_ were used instead of the corresponding, commercially available, but much more expensive, pre-prepared QuEChERS extraction kits. In our experiments, the usage of bulk materials produced comparable results. 270 mg solid phase extraction (SPE) material used equals roughly one centrifuge tube containing the Agilent Dispersive 2 mL Universal kit #5982-0028CH consisting of 50 mg primary secondary amine (PSA), 50 mg C_18_EC, 7.5 mg Bulk Carbograph, and 150 mg MgSO_4_. After evaporation, samples were stored at −20 °C until the analysis *via* LC-ESI/MS. This allowed the preparation of large amounts of samples as well as the measurement in a continuous sequence in order to minimize instrument specific variance and ensure comparability of the resulting data. After resuspension in 1 mL acetonitrile/ddH_2_O (50/50 vol.%), a final filtration step was implemented to prevent the injection of any residues of the SPE-material or parts of the organic matrix that could potentially influence or even damage the LC-ESI/MS system. Polytetrafluorethylene (PTFE) filters (0.2 μm) with a diameter of 13 mm (low dead-volume) were used to minimize the loss of sample volume. Illustrations of key extraction steps are shown in Fig. 2.

### LC-ESI/MS analytics

Thiacloprid and flupyradifurone were analyzed using an Agilent 1260 Infinity Series High-Performance Liquid Chromatography system (HPLC, Agilent Technologies Sales & Services GmbH & Co. KG, Waldbronn, Germany). The LC-ESI/MS system consisted of a binary pump and an ESI single-quadrupole MS detector (Agilent 6130). Software OpenLab CDS for LC–MS (Rev. C.01.07-SP3) was used for the chromatographic analysis. The HPLC method was performed on the reversed-phase column Agilent XDB C_18_ (150 x 4.6 mm; 5 μm, Agilent Technologies Sales & Services GmbH & Co. KG, Waldbronn, Germany). The system was operated in gradient mode at a temperature of 45 °C with a flow rate of 0.75 mL/min. The mobile phase consisted of 65% ddH_2_O with 0.1% formic acid (solvent A) and 35% acetonitrile (solvent B). A linear gradient according to Table 1 was used:

**Table 1:** LC gradient.

Time [min]	Solvent A [%]	Solvent B [%]
0.00	65	35
4.50	62	38
5.00	35	65
8.00	35	65
8.01	65	35
14.00	65	35

Detection was performed using an ESI/MS detector. Gas temperature 350 °C; drying gas flow 12.5 L min^−1^; nebulizer pressure 45 psig; V_cap_ +4000 V, single ion mode (SIM) thiacloprid: *m/z* 253, 254, 255, SIM flupyradifurone: *m/z* 289, 290, 291. The injection volume was set to 1 μL. Standards of thiacloprid and flupyradifurone ranging from 1 to 20 ng L^−1^ and 5 to 200 ng L^−1^, respectively, were used for calibration. All standards and samples were analyzed in triplicates.

## Method validation

**Fig. 3:**
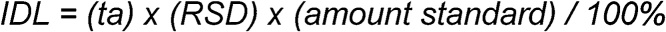
The equation for the instrument detection limit (IDL).

The method was validated by calculating the limit of detection (LOD) and limit of quantification (LOQ) by calculation of the instrument detection limit (IDL) according to following equation (Fig. 3). In this context, calibration curve measurements were performed in triplicates for both thiacloprid (THIA) and flupyradifurone (FLUPY) with a confidence factor of 99%. Linear ranges were established using calibration curves that were constructed in the typical concentration ranges for each particular compound (Table 2). The recovery rate was ascertained using three different concentrations.

**Table 2:** Method validation data.

Substance	Linear range (ng mL^−1^)	Linear regression *(R*^2^)	IDL aStD (ng mL^−1^) (n = 3)	Cross validation (CV %) (n = 3)
Flupyradifurone	5 - 200	0.9938	3.1 (10 ng mL^−1^)	4.5 (10 ng mL^−1^)
Thiacloprid	1 - 20	0.9998	1 (10 ng mL^−1^)	1.5 (10 ng mL^−1^)

^1^Concentrations at which CV was assessed are shown in brackets.

a StD = standard deviation.

### Recovery

To test the robustness of our presented extraction, purification and analytical method, recovery experiments were investigated. Three different concentrations were tested. The recovery rate for the low concentration was less than 50%, however, this concentration proved to be below the established LOQ for THIA. For the two higher concentrations, which are within the range of quantification (above LOQ), the average recovery rate was between 70 and 80%. The reproducibility, presented as standard deviation, between ±3 – 6% was satisfying over the entire range of concentrations tested (Table 3).

**Table 3:** Summary of method validation parameters.

Substance	LOD (ng g^−1^)	LOQ (ng g^−1^)	Average recovery (%) (n = 3)	Cross validation (CV %) recovery (n = 3)
Flupyradifurone	19.72	65.73	–	–
Thiacloprid	7.61	25.36	64.3	6 (0.6 μg mL^−1^)

^1^Concentrations at which CV was assessed are shown in brackets. Area values are listed in supplementary table S3.

### Honey bee sampling

Bee samples from four different beehives were provided from collaboration partners specialized in honey bee field studies. No information about thiacloprid or flupyradifurone contamination was known. The developed method was applied to all of the samples. Table 4 shows exemplary the results of two polluted honey bee samples. The bees were extracted, and the supernatant was purified and analyzed according to our protocol. No cross contaminations could be monitored. Chromatograms still display some matrix effects, but at an acceptable level to get distinguishable and analyzable peaks for both pesticides. The tests based on real bee samples/matrix further prove the applicability of the developed method, and the results were in accordance with the field studies.

**Table 4:** Exemplary results of two independent field study samples.

Sample	Substance	aConc. 1 [ng g^−1^]	Conc. 2 [ng g^−1^]	Conc. 3 [ng g^−1^]	bStD [ng g^−1^]	Average concentration [ng g^−1^]	CV (%) (n = 3)
1	Thiacloprid	227.30	232.57	217.54	7.63	225.80	3.4
2	Flupyradifurone	263.83	187.74	324.74	68.64	258.77	26.5

a conc. = concentration, Area values are listed in supplementary table S4.

b StD = standard deviation.

## Additional information

In the last years, great concern about the impact of a standard class of pesticides, known as neonicotinoids, on honey bees (*Apis mellifera*) and other native pollinators has been raised. Neonicotinoids are systemic insecticides that are taken up by plant leaves as well as roots and provide very effective control of piercing and sucking by insects. Until now, neonicotinoids are used in hundreds of different insecticides [[1], [2], [3]].

One widespread molecule used in insecticides is thiacloprid (THIA). THIA is similar to the natural insecticide nicotine, which acts on the central nervous system as an agonist of the acetylcholine receptor. THIA can mimic the action of the neurotransmitter that naturally occurs in the insect body, acetylcholine (ACh), on some of its neuronal receptors (known as ‘nicotinic receptors’). It is reported that thiacloprid exposure can lead to changes in the honey bees sense of smell and habituation and subsequent disorientation effects [4,5].

**Fig. 4:**
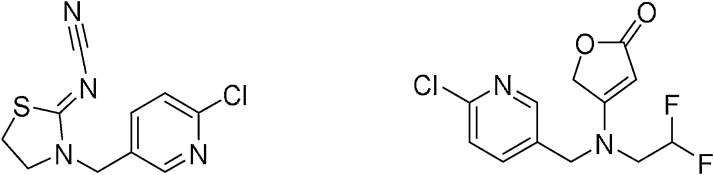
Target molecules. Thiacloprid ((*Z*)-[3-[(6-chloro-3-pyridinyl)methyl]-2-thiazolidinylidene]cyanamid, left structure) *m/z* 252.7, flupyradifurone ((4-[(2,2-difluoroethyl)amino]-2(5*H*)-furanone, right structure) *m/z* 288.7.

In parallel, a new chemical insecticide for efficient crop protection measures was developed (Fig. 4). The butenolide scaffold in naturally-occurring stemofoline inspired researchers for flupyradifurone (FLUPY) development. The pesticide acts reversibly as an agonist on insect nicotinic acetylcholine receptors. It is structurally different from known agonists, as shown by chemical similarity analytics. The molecule might become a new agrochemical to substituted forbidden neonicotinoids all over the world [[6], [7], [8]].

Many scientific groups all over the world are working on this severe and complex problem by identifying THIA and FLUPY concentrations in different matrices. In this context, many different chromatographic methods are described [9,10]. Traditionally, pesticide residues were analyzed using physicochemical techniques, especially by liquid chromatography (LC)-coupled with tandem mass spectrometry (MS/MS). By the end of the 20th century, LC—MS had evolved dramatically as a primary analytical tool providing both sufficient sensitivity and selectivity.

Furthermore, LC–MS is versatile enough to be used either as a screening tool or as a quantitative method (or both), depending on the application. The use of LC–MS is now a common practice, and the technique is continuously updated to improve its sensitivity and selectivity. The analysis of multiple-analyte traces in varying matrices is of great interest, and many different methods have been developed.

The most common strategy is to couple a liquid or gas-chromatography separation technique with a UV/vis absorbance or fluorescence-detection system. In parallel, state of the art for the detection of pesticides is the usage of an LC–MS/MS or Q-TOF system [[11], [12], [13]].

However, not every scientific group or smaller company has unlimited access and experience to use this very sensitive, but expensive and sophisticated analytical technique. Due to the fact that the usage, influence, and consequence of neonicotinoids is a global challenge, a robust analytical method based on cheaper equipment is essential. This is especially true for research groups for developing countries with less financial support. To overcome this hurdle and to open new possibilities for a broader research community, in some cases, the usage of a cheaper single-quadrupole MS detector represents an opportunity.

Unfortunately, matrix components can co-elute and influence reproducibility and accuracy using a single-quadrupole MS. To diminish some of these limitations, an appropriate sample pre-preparation is essential to achieve high sensitivity, reproducibility, and less system contamination [14]. In a complete analytical procedure, sample pre-preparation, including a clean-up and/or concentration step, is still the most time- as well as lab-consuming step.

The simplest method is to apply a solid-liquid extraction and filtration step to avoid particles. The drawback is that all soluble molecules can cause drastic increase of the background/matrix. A very effective way to minimize unwanted effects is the usage of solid-phase (SPE) or solid-liquid extraction (SLE). Several solid materials, in different volumes, in ready to use cartridges, are commercially available. In this case, the eluent is matrix-minimized, but the cleaning procedure still needs time and experience. A promising dispersive method (dSPE) combining these two techniques is the already widespread QuEChERS (Quick, Easy, Cheap, Effective, Rugged, and Safe) sample cleaning method, combining efficiency and promising results [[15], [16], [17]]. Especially the purification of many samples and the possibility to use kits are convincing arguments for QuEChERS. dSPE reduces extraction time and costs, and avoids excess amounts of solvent, waste, glassware. Moreover, minimal training is required compared to classical SPE.

Therefore, we developed and applied a fast method to determine the concentration of thiacloprid and flupyradifurone in honey bees by a single-quadrupole LC-ESI/MS based on a modified QuEChERS sample pre-preparation procedure to reduce influencing matrix effects.
