# Supplementary material for: A simple and robust LC-ESI single quadrupole MS-based method to analyze neonicotinoids in honey bee extracts
Source: MethodsX. 2019 Oct 17;6:2484–91. doi: 10.1016/j.mex.2019.09.038 (PMC6838890; doi:10.1016/j.mex.2019.09.038)
Supplement: Supplementary file 1 [file mmc1.docx]

**Supplementary material**

**Calibration curve**

**Table S1.** LC-MS calibration curve data for thiacloprid.

| Thiacloprid | Concentration  [ng mL^-1^] | Area 1 | Area 2 | Area 3 | Average  area | *StD |
| --- | --- | --- | --- | --- | --- | --- |
| blank | 0 | 0 | 0 | 0 | 0 | 0 |
| 1 | 1 | 8100 | 7950 | 8020 | 8023 | 75 |
| 2 | 2 | 9631 | 13758 | 12031 | 11806 | 2072 |
| 3 | 5 | 28904 | 27778 | 27898 | 28193 | 618 |
| 4 | 10 | 52750 | 54199 | 52960 | 53303 | 783 |
| 5 | 20 | 108509 | 106238 | 97902 | 104216 | 5585 |

*StD standard deviation

**Table S2.** LC-MS calibration curve data for flupyradifurone.

| Flupyradifurone | Concentration  [ng mL^-1^] | Area 1 | Area 2 | Area 3 | Average  area | *StD |
| --- | --- | --- | --- | --- | --- | --- |
| blank | 0 | 0 | 0 | 0 | 0 | 0 |
| 1 | 5 | 19924 | 19332 | 22214 | 20490 | 1522 |
| 2 | 10 | 38333 | 41364 | 41559 | 40418 | 1808 |
| 3 | 20 | 73500 | 73248 | 71883 | 72877 | 870 |
| 4 | 100 | 392798 | 389612 | 397301 | 393237 | 3863 |
| 5 | 200 | 664449 | 684677 | 684966 | 678030 | 11763 |

*StD standard deviation


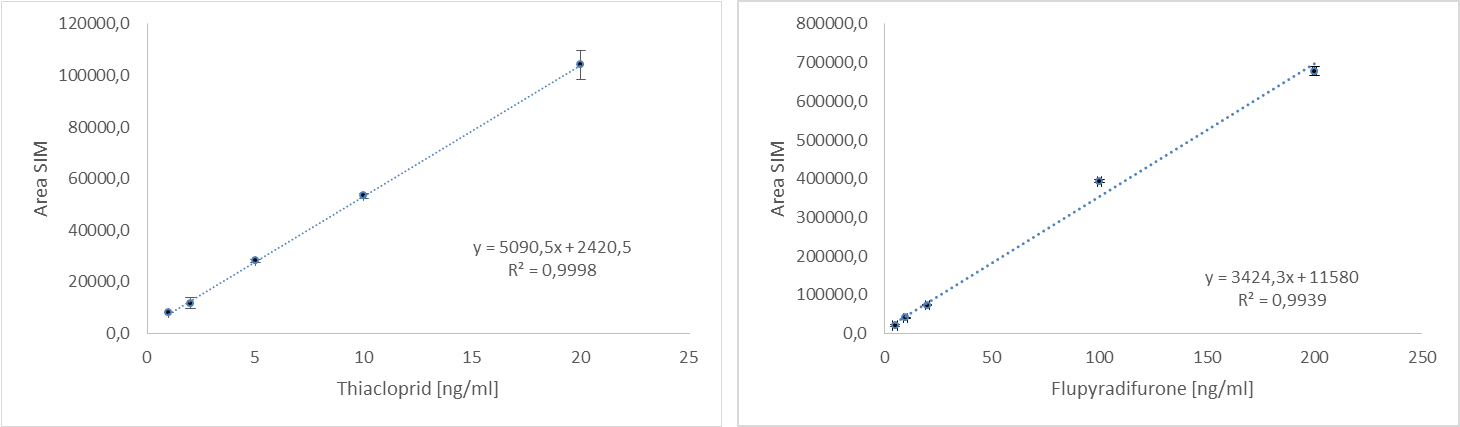


**Fig. S1.** Calibration curves and linear range of thiacloprid and flupyradifurone 2 – 20 ng mL^-1^ and 5 – 200 ng mL^-1^. Each concentration was investigated in triplicates. All standards were dissolved in 50% acetonitrile / 50% *dd*H_2_O. Calibration curve data table S1 and S2.


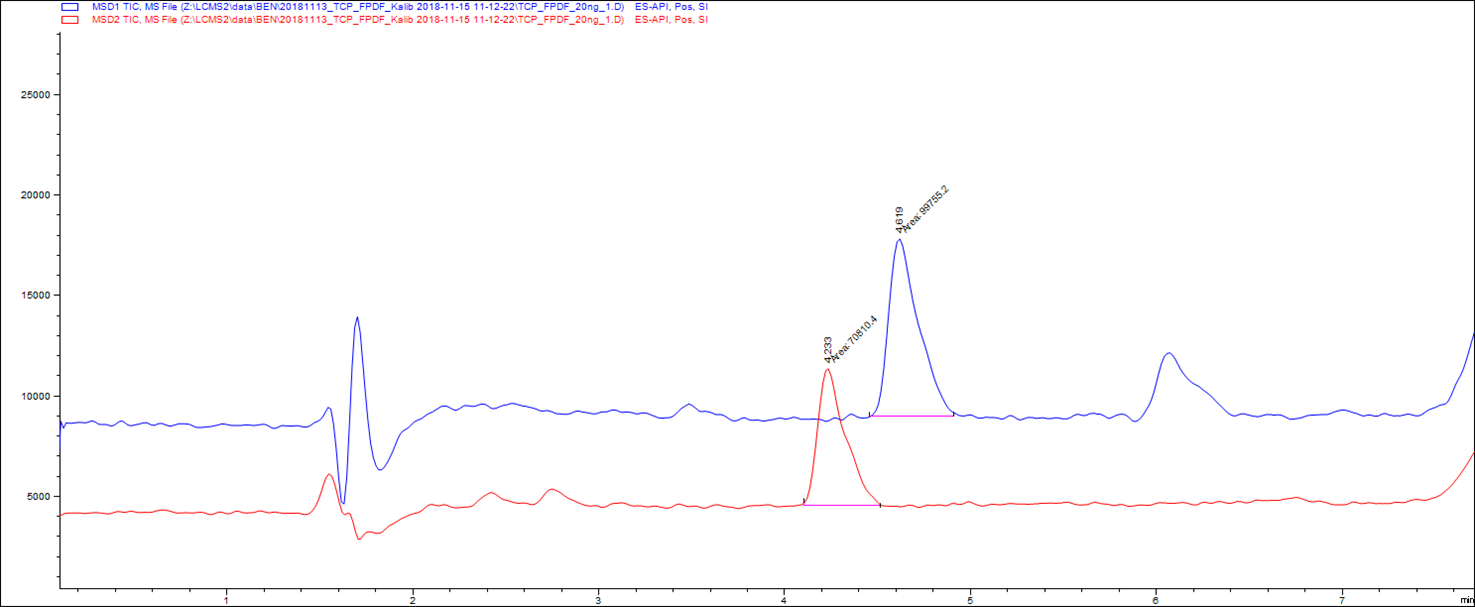


**Fig. S2.** LC-MS chromatogram of A) flupyradifurone in red (rt 4.23 min) measured in SIM mode at m/z 289, 290, 291 and B) thiacloprid in blue (rt 4.62 min) measured in SIM mode at m/z 253, 254, 255.

**Table S3.** Recovery rate of thiacloprid spiked honeybee samples after QuEChERS purification.

| **Sample** | **Expected Area** | **Observed Area** | **Zero adjustment** | **Recovery [%]** | **Average Recovery [%]** | ***StD** |
| --- | --- | --- | --- | --- | --- | --- |
| 0.01 µg mL^-1^_1 | 31621 | 31943 | 14609 | 46 | 40 | ± 6 |
| 0.01 µg mL^-1^_2 | 31621 | 28157 | 10824 | 34 |  |  |
| 0.01 µg mL^-1^_3 | 31621 | 30191 | 12857 | 41 |  |  |
| 0.60 µg mL^-1^_1 | 1264311 | 990983 | 973649 | 77 | 81 | ± 6 |
| 0.60 µg mL^-1^_2 | 1264311 | 1133559 | 1116225 | 88 |  |  |
| 0.60 µg mL^-1^_3 | 1264311 | 1000202 | 982868 | 78 |  |  |
| 1.00 µg mL^-1^_1 | 1875571 | 1359325 | 1341992 | 72 | 72 | ± 3 |
| 1.00 µg mL^-1^_2 | 1875571 | 1300676 | 1283341 | 68 |  |  |
| 1.00 µg mL^-1^_3 | 1875571 | 1431432 | 1414099 | 75 |  |  |

*StD standard deviation


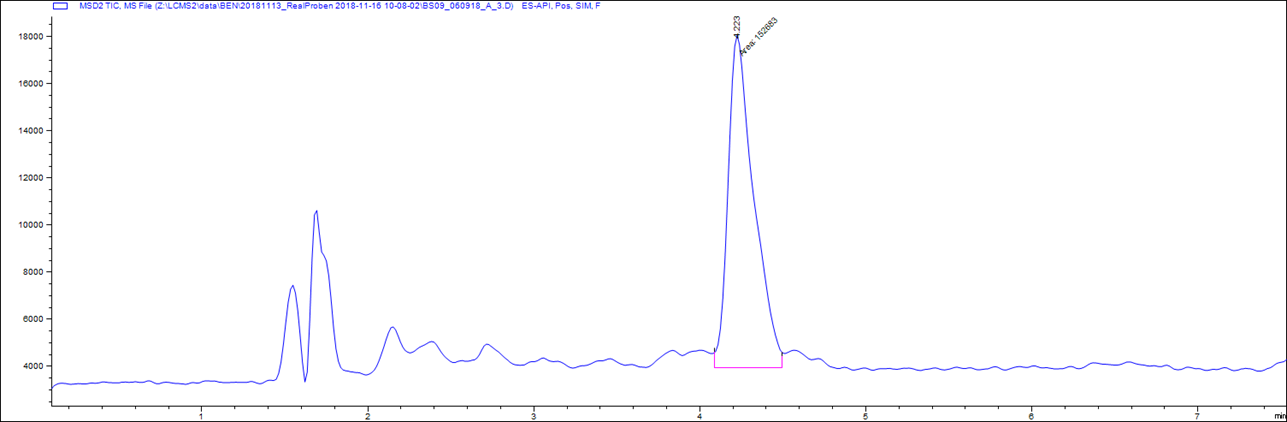


**Figure S3.** LC-MS chromatogram of a honey bee sample fed with flupyradifurone (rt 4.23 min) after sample preparation according to figure 1. Measured in SIM mode at m/z 289, 290, 291.


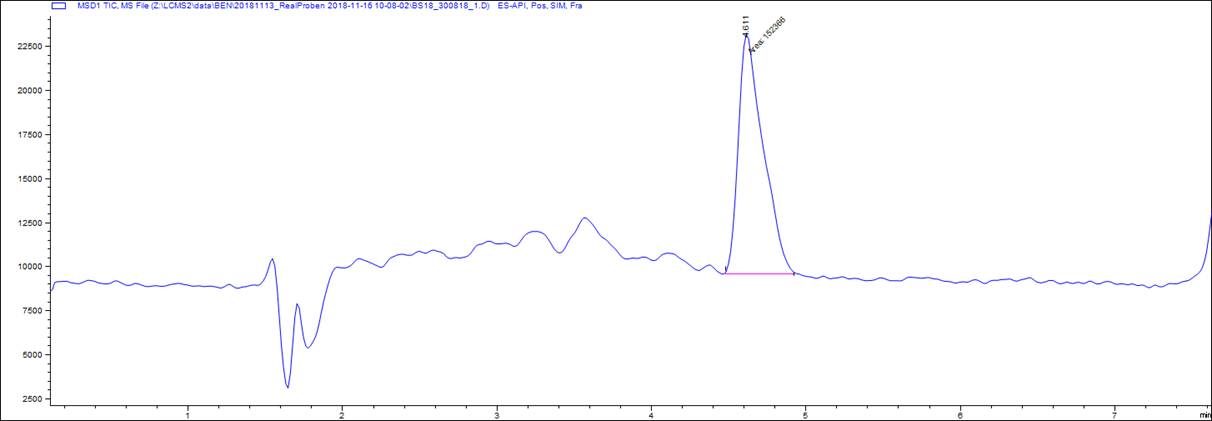


**Figure S4.** LC-MS chromatogram of a honey bee sample fed with thiacloprid (rt 4.62 min) after sample preparation according to figure 1. Measured in SIM mode at m/z 253, 254, 255.

**Table S4.** Raw data for the two independent field study samples.

|  | Initial weight [mg] | Area SIM | Concentration  [ng g^-^1] |
| --- | --- | --- | --- |
| THIA / 1 | 129.2 | 151896 | 227.30 |
| THIA / 2 | 165.2 | 197980 | 232.57 |
| THIA / 3 | 150.4 | 168954 | 217.54 |
| FLUPY / 1 | 109.7 | 110685 | 263.83 |
| FLUPY / 2 | 156.0 | 111867 | 187.74 |
| FLUPY / 3 | 123.4 | 148802 | 324.74 |
