# Supplementary material for: A simple and robust LC-ESI single quadrupole MS-based method to analyze neonicotinoids in honey bee extracts
Source: MethodsX. 2019 Oct 17;6:2484–91. doi: 10.1016/j.mex.2019.09.038 (PMC6838890; doi:10.1016/j.mex.2019.09.038)
Supplement: Supplementary file 2 [file mmc2.docx]

**Table S1.** LC-MS calibration curve data for thiacloprid.

| Thiacloprid | Concentration  [ng mL^-1^] | Area 1 | Area 2 | Area 3 | Average  area | *StD |
| --- | --- | --- | --- | --- | --- | --- |
| blank | 0 | 0 | 0 | 0 | 0 | 0 |
| 1 | 1 | 8100 | 7950 | 8020 | 8023 | 75 |
| 2 | 2 | 9631 | 13758 | 12031 | 11806 | 2072 |
| 3 | 5 | 28904 | 27778 | 27898 | 28193 | 618 |
| 4 | 10 | 52750 | 54199 | 52960 | 53303 | 783 |
| 5 | 20 | 108509 | 106238 | 97902 | 104216 | 5585 |

*StD standard deviation
