# Supplementary material for: A simple and robust LC-ESI single quadrupole MS-based method to analyze neonicotinoids in honey bee extracts
Source: MethodsX. 2019 Oct 17;6:2484–91. doi: 10.1016/j.mex.2019.09.038 (PMC6838890; doi:10.1016/j.mex.2019.09.038)
Supplement: Supplementary file 3 [file mmc3.docx]

**Table S2.** LC-MS calibration curve data for flupyradifurone.

| Flupyradifurone | Concentration  [ng mL^-1^] | Area 1 | Area 2 | Area 3 | Average  area | *StD |
| --- | --- | --- | --- | --- | --- | --- |
| blank | 0 | 0 | 0 | 0 | 0 | 0 |
| 1 | 5 | 19924 | 19332 | 22214 | 20490 | 1522 |
| 2 | 10 | 38333 | 41364 | 41559 | 40418 | 1808 |
| 3 | 20 | 73500 | 73248 | 71883 | 72877 | 870 |
| 4 | 100 | 392798 | 389612 | 397301 | 393237 | 3863 |
| 5 | 200 | 664449 | 684677 | 684966 | 678030 | 11763 |

*StD standard deviation
