# Supplementary material for: A simple and robust LC-ESI single quadrupole MS-based method to analyze neonicotinoids in honey bee extracts
Source: MethodsX. 2019 Oct 17;6:2484–91. doi: 10.1016/j.mex.2019.09.038 (PMC6838890; doi:10.1016/j.mex.2019.09.038)
Supplement: Supplementary file 4 [file mmc4.docx]

**Table S3.** Recovery rate of thiacloprid spiked honeybee samples after QuEChERS purification.

| **Sample** | **Expected Area** | **Observed Area** | **Zero adjustment** | **Recovery [%]** | **Average Recovery [%]** | ***StD** |
| --- | --- | --- | --- | --- | --- | --- |
| 0.01 µg mL^-1^_1 | 31621 | 31943 | 14609 | 46 | 40 | ± 6 |
| 0.01 µg mL^-1^_2 | 31621 | 28157 | 10824 | 34 |  |  |
| 0.01 µg mL^-1^_3 | 31621 | 30191 | 12857 | 41 |  |  |
| 0.60 µg mL^-1^_1 | 1264311 | 990983 | 973649 | 77 | 81 | ± 6 |
| 0.60 µg mL^-1^_2 | 1264311 | 1133559 | 1116225 | 88 |  |  |
| 0.60 µg mL^-1^_3 | 1264311 | 1000202 | 982868 | 78 |  |  |
| 1.00 µg mL^-1^_1 | 1875571 | 1359325 | 1341992 | 72 | 72 | ± 3 |
| 1.00 µg mL^-1^_2 | 1875571 | 1300676 | 1283341 | 68 |  |  |
| 1.00 µg mL^-1^_3 | 1875571 | 1431432 | 1414099 | 75 |  |  |

*StD standard deviation
