# Supplementary material for: A simple and robust LC-ESI single quadrupole MS-based method to analyze neonicotinoids in honey bee extracts
Source: MethodsX. 2019 Oct 17;6:2484–91. doi: 10.1016/j.mex.2019.09.038 (PMC6838890; doi:10.1016/j.mex.2019.09.038)
Supplement: Supplementary file 5 [file mmc5.docx]

**Table S4.** Raw data for the two independent field study samples.

|  | Initial weight [mg] | Area SIM | Concentration  [ng g^-^1] |
| --- | --- | --- | --- |
| THIA / 1 | 129.2 | 151896 | 227.30 |
| THIA / 2 | 165.2 | 197980 | 232.57 |
| THIA / 3 | 150.4 | 168954 | 217.54 |
| FLUPY / 1 | 109.7 | 110685 | 263.83 |
| FLUPY / 2 | 156.0 | 111867 | 187.74 |
| FLUPY / 3 | 123.4 | 148802 | 324.74 |
